# Acoustic Property Reconstruction of a Neonate Yangtze Finless Porpoise's (*Neophocaena asiaeorientalis*) Head Based on CT Imaging

**DOI:** 10.1371/journal.pone.0121442

**Published:** 2015-04-09

**Authors:** Chong Wei, Zhitao Wang, Zhongchang Song, Kexiong Wang, Ding Wang, Whitlow W. L. Au, Yu Zhang

**Affiliations:** 1 Key Laboratory of Underwater Acoustic Communication and Marine Information Technology (Xiamen University), Ministry of Education, Xiamen 361005, Fujian, China; 2 Hawaii Institute of Marine Biology, University of Hawaii, 46–007 Lilipuna Road, Kaneohe, Hawaii 96744, United States of America; 3 Key Laboratory of Aquatic Biodiversity and Conservation of the Chinese Academy of Sciences, Institute of Hydrobiology of the Chinese Academy of Sciences, Wuhan 430072, P. R. China; 4 University of Chinese Academy of Sciences, Beijing 100039, P. R. China; 5 College of Oceanography and Environmental Science, Xiamen University, Xiping Building, Xiangan South Road, Xiamen, 361005, P. R. China; 6 College of Ocean & Sciences, Xiamen University, Xiamen 361005, Fujian, China; Texas Christian University, UNITED STATES

## Abstract

The reconstruction of the acoustic properties of a neonate finless porpoise’s head was performed using X-ray computed tomography (CT). The head of the deceased neonate porpoise was also segmented across the body axis and cut into slices. The averaged sound velocity and density were measured, and the Hounsfield units (HU) of the corresponding slices were obtained from computed tomography scanning. A regression analysis was employed to show the linear relationships between the Hounsfield unit and both sound velocity and density of samples. Furthermore, the CT imaging data were used to compare the HU value, sound velocity, density and acoustic characteristic impedance of the main tissues in the porpoise’s head. The results showed that the linear relationships between HU and both sound velocity and density were qualitatively consistent with previous studies on Indo-pacific humpback dolphins and Cuvier’s beaked whales. However, there was no significant increase of the sound velocity and acoustic impedance from the inner core to the outer layer in this neonate finless porpoise’s melon.

## Introduction

The Yangtze finless porpoise (*Neophocaena asiaeorientalis*) is listed as critically endangered on the International Union for the Conservation of Nature Red List of Threatened Species [1]. They are distributed in the main stem of the middle and lower reaches of the Yangtze River and in the adjoining Poyang and Dongting Lakes [2]. Finless porpoises stay mainly in shallow offshore waters and use their biosonar systems for foraging, group communication, and avoiding predators. There have been some studies investigating sound production and acoustic signal characteristics [3–9].

The function of the melon has been experimentally studied by Jing et al. [10] and they concluded that the melon of the bajii (*Lipotes vexillifer*) played an important role in the beam formation. Hua et al. [11] have sliced the melon of a bajii to measure the ultrasonic attenuation and sound velocity. Computed tomography (CT) scanned technology has also been widely used for modeling the forehead of odontocetes. Aroyan et al. [12, 13] have used CT imaging to build two-dimensional and three-dimensional models for numerically simulating the biosonar beam formation and hearing in the *Delphinus delphis*. Krysl et al. [14, 15] have studied the vibration of biosolids immersed in fluids and the effect of high-intensity sound on *Ziphius cavirostris* based on the CT scan technology. Cranford et al. [16–18] have investigated the sound production and hearing processes of *Ziphius cavirostris* and how the biosonar beam is focused in the forehead of *Tursiops truncatus*. Soldevilla et al. [19] found a linear relationship between both the sound velocity and density of the tissues with the Hounsfield unit (HU) obtained from CT scanning of the Cuvier’s beaked whale. The acoustic properties of the forehead of the finless porpoise have not been investigated. Because the finless porpoise is a critically endangered species, it is important to learn as much as we can about sound production and hearing in this species.

In this paper, CT images will be used to determine the HU distribution in a neonate porpoise head. The relationships between the HU value and both sound velocity and density values will be obtained for reconstruction. HU value, sound velocity, density and acoustic characteristic impedance of the main tissues (blubber, connective tissue, muscle, melon, and mandibular fat) in the porpoise’s head will be statistically compared.

## Materials and Methods

A neonate porpoise was stranded and rescued for rehabilitation on May 21, 2013 by the Institute of Hydrobiology, Chinese Academy of Sciences. The porpoise had a body length of 69 cm and seemed to be less than one month old. Unfortunately, it died on the next day and its body was immediately frozen to avoid decomposition. CT scan, sound velocity and density measurements were performed on the carcass. The research was carried out under a research permit issued to the Institute of Hydrobiology of the Chinese Academy of Sciences by the Ministry of Agriculture of China. The approval number from the Fishery Bureau of the General Office of the Ministry of Agriculture was 2012–114.

### CT Scan

**Fig 1:**
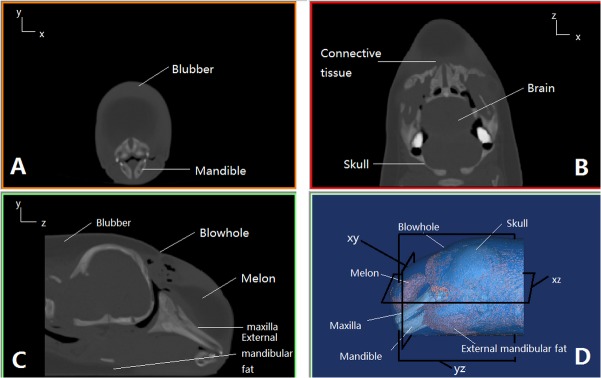
The 2-D and 3-D views of the finless porpoise’s head. (A) The axial cross section (xy, front view). (B) coronal cross section (xz, top view). (C) sagittal cross section (yz, lateral view). (D) 3-D reconstructed graph of the finless porpoise’s head by CT scan.

The porpoise was scanned at the Zhongnan Hospital of Wuhan University on May 23, 2013. Computed tomography scans were conducted on a SOMATOM Definition Dual Source CT (DSCT; Siemens, Germany) with 1 mm slice width. The images were collected at a power setting of 120kV×76 mA and saved on a hard drive as a matrix of 512 x 512 pixels per image in the IMA format. Using multiple projections of the CT images, the internal structures of the porpoise were reconstructed. The CT numbers were derived by comparing the linear attenuation coefficient of a pixel with that of water which was described in Hounsfield units. CT scan data were analyzed to determine HU values of tissue samples. The range of the HUs were scaled from—1000 to >1000, where air was—1000, room temperature water was 0, and HU greater than 1000 was considered as calcified bone [20]. The general range of the mammalian soft tissues was between—100 and 100 with fatty tissues at the low end and denser connective tissues at the high end [21]. The longitudinal axis of the body was considered the z-axis, the cross sectional plane of the porpoise (across body axis) as the x-axis, and the sagittal plane of the porpoise as the y-axis. Thus the axial cross section (xy plane), coronal cross section (xz plane) and sagittal cross section (yz plane) were obtained and are shown in Fig 1A–1C, respectively, where the main anatomical structures, such as blubber, connective tissue, melon, skull, brain, mandible, and external mandibular fat are indicated. A three-dimensional model of the porpoise’s head was reconstructed using the software Mimics 10.1 (Materialise, Belgium) and is shown in Fig 1D, where three frames represent the three sections in Fig 1A–1C, the gray regions represent the bony structures including maxilla, mandible, and skull, and the red regions represent the soft tissue including melon and external mandibular fat.

### Sound Velocity Measurement

**Fig 2:**
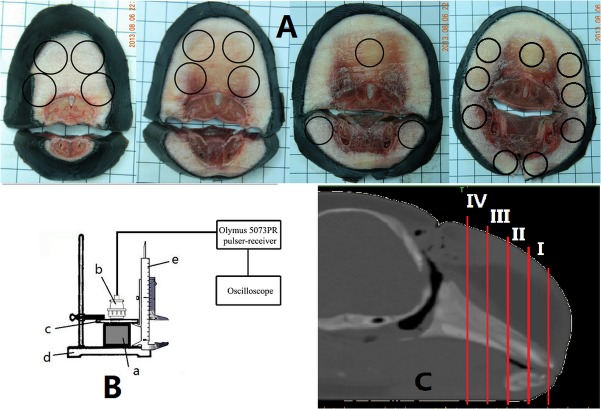
Schematic diagram of the measurement. (A) The four sections (I, II, III, IV) which are transverse to the body axis and the positions where sound velocity measurement were taken. (The circles represent the positions where the probe was put). Because the slices are not the same size (the slice is getting bigger from I~IV), different magnification was used for better illustration of these slices. (B) The diagram of the measurement instrument (a. slices; b. Olymus 5073PR ultrasonic pulser-receiver probe; c. plexiglass; d: bottom metal baffle-board, e. vernier caliper). (C) The position where each section is from.

**Fig 3:**
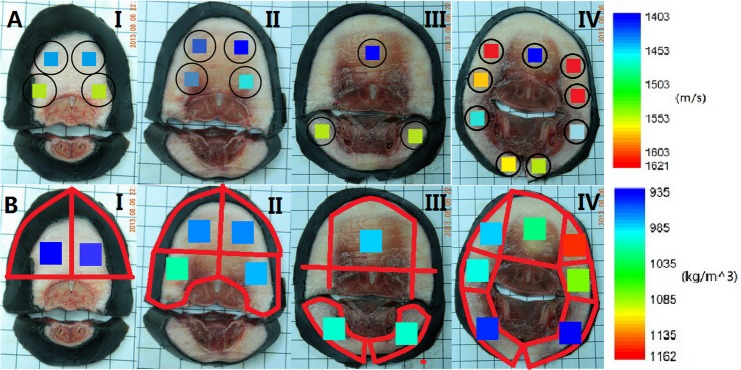
The measured sound velocities and densities of the tissue samples cut from four transverse slices. (A) The measured sound velocities of the tissue samples cut from four transverse slices. (B) The measured densities of the tissue samples cut from four transverse slices. The images I to IV in both figures correspond to anterior to posterior sides of the finless porpoise head (the dorsal side is up and the background grids are 1 cm×1 cm).

The specimen was sliced transversely across the body axis from anterior to posterior with approximately 1 cm width (see Fig 2C) when the specimen was still frozen. The four slices (I, II, III and IV) were kept frozen in the refrigerator under the temperature of 0°C. Each slice was cut into several small samples according to the red lines in Fig 3B for the tissue measurements. After the samples were completely thawed, the density and sound velocity measurements were performed. Sound velocity measurement of the porpoise’s head tissues was performed by using an ultrasound velocimeter. In order to avoid any influence from temperature changes, every slice was individually taken for the measurements to a laboratory room where the temperature remained at 23°C. The density measurement was performed immediately after the sound velocity measurement. Sixteen total samples were used for the density measurement and 25 samples for the sound velocity measurement. Some of slices were measured on both face and back sides to make sure that the measurement error would be low enough, which caused the number of samples of sound velocity measurement results to be greater than those of density. Then, each sample was put on a smooth metal baffle-board with a fine reflection effect and a plexiglass sheet placed parallel to the baffle-board was used to fix the sample. The small samples were placed naturally on the baffle-board without significant shape changing. The plexiglass was controlled by a button to go down carefully to avoid changing the shape of the samples. The same procedure was repeated five times. The measured sound velocity did not show significant fluctuation. This suggests that there might not have been significant tissue compression to influence sound velocities.

An Olympus 5073PR ultrasonic pulser-receiver with the probe diameter of 1.5 cm was used to produce the ultrasound pulse; the frequency of the pulse was set as 3.5 MHz to ensure the accuracy of the time measurement. The positions where the probe was put are represented as circles in Fig 2A. Some of the ultrasound pulses were first reflected by the plexiglass surface, causing the first reflected wave received by the probe. Then, the other ultrasound pulses further propagated through plexiglass and the test sample then were reflected by the bottom metal baffle board, resulting in a second reflected wave. The time difference Δ*t*, between the first and second reflected tonal pulses was measured with an oscilloscope. The distance *d* between the bottom metal baffle and the plexiglass was measured with a vernier caliper, as shown in the diagram of Fig 2B. Thus the sound velocity was determined as:

$$\bar{c}=2d/\Delta t$$ 1

The time difference Δ*t* and distance *d* of each sample were measured 5 times to obtain their averaged values.

### Density Measurement

An electronic balance accurate to 0.001 g was used for mass measurements of the small tissue samples which were cut according to the red lines in Fig 3B. The mass of each small sample was measured five times to calculate the mean value $\bar{m}$.

Water was then added into a cylinder with the accuracy of 1 ml and the volume *V*′, was measured. A glass rod was attached to the tissues during the density measurement. The effective density of the tissue attached to the glass rod was greater than that of water to ensure it would immerse into the water of the measurement cylinder, creating the volume *V*_1_. The volume of the glass rod *V*_*g*_ was also measured. Thus the volume difference Δ*V* was *V*_1_−*V*_*g*_−*V*′. After the first measurement, the samples remained above the cylinder until all water droplets dropped back the cylinder so that the residual liquid volume was negligible compared with the tissue volume. The sample was then immersed into the water to repeat the measurement, obtaining a new volume *V*_2_. This process was repeated five times for each sample, giving the averaged value of the volume difference $\Delta \bar{V}$. From the measured $\bar{m}$ and $\Delta \bar{V}$, using Archimedes’ principle, the density of the sample then was determined as:

$$\bar{\rho }=\bar{m}/\Delta \bar{V}$$ 2

Based on the sound velocity and density measurements, the acoustic character impedance *Z*_*s*_ of each tissue sample was derived as:

$$Z_{s}=\rho c$$ 3

### Statistical Analysis

A regression analysis was performed to determine the relationships between HU and sound velocity as well as density. Soldevilla et al. [19] found that the sound velocity and density of the Cuver’s beaked whale’s forehead tissues were linearly related to the HU values. A univariate regression analysis was used to obtain the linear regression equations of HU with sound velocity and density. Based on these linear regression equations, the distributions of sound velocity, density and acoustic impedance at the coronal and sagittal cross sections were reconstructed. Because the tested groups may not be from normally distributed populations with equal variances, the Kruskal-Wallis one-way analysis of variance (ANOVA) on ranks was employed using HU, sound velocity, density, and acoustic impendence as dependent variables and the tissue types (blubber, connective tissue, muscle, melon, and mandibular fat) as independent variables. The statistical significance level was set as 0.05. ANOVAs were followed up with a post hoc Dunn’s test that was used for multiple comparisons of groups to determine which groups were significantly different. SigmaStat 3.0 and SigmaPlot 8.0 (Jandel Scientific, SanRafael, CA) were used to statistically analyze and graph the results.

## Results

The measured sound velocities and densities of the tissue samples cut from 4 transverse slices are shown in Fig 3, where the images from slice I to IV correspond to anterior to posterior side of the finless porpoise’s head with the dorsal side up. The values of the sound velocity and density values can be estimated with the color scale. The circles in Fig 3A represent the measured positions of sound velocity where the ultrasound probe was placed and the colored areas represent the sound velocity values based on the color scale to the right side of the figure. Low sound velocity regions associated with melon tissue are shown. The colored areas in Fig 3B represent the density values of the small samples which were cut according to the red lines. The low-density regions are also found in melon tissue. To derive the relationships between HU values and sound velocity, the averaged HU values of the corresponding regions in the sound velocity measurement were calculated. The same procedure was also performed for the relationship between density and HU values.

**Fig 4:**
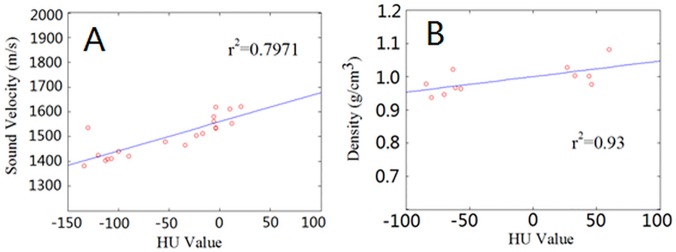
Regression analysis of HU vs. sound velocity and density for the finless porpoise forehead tissues. (A) Regression analysis of HU vs. sound velocity for the finless porpoise forehead tissues. (B) Regression analysis of HU vs. density for the finless porpoise forehead tissues.

The relationship between HU values and both sound velocity and density of the tissues in the head are shown in Fig 4. The univariate regression analysis showed a linear correlation between sound velocity and HU (*r* ^2^ = 0.80), in Fig 4A, where the linear regression equation is given by the equation

$$\bar{c}=1.1773\times \bar{HU}+1501.4$$ 4

Similarly, the regression analysis found that density and HU were linearly related (*r* ^2^ = 0.93) as shown in Fig 4B, where the linear regression equation is given by the equation

$$\bar{\rho }=0.0005\times \bar{HU}+1.0008$$ 5

**Fig 5:**
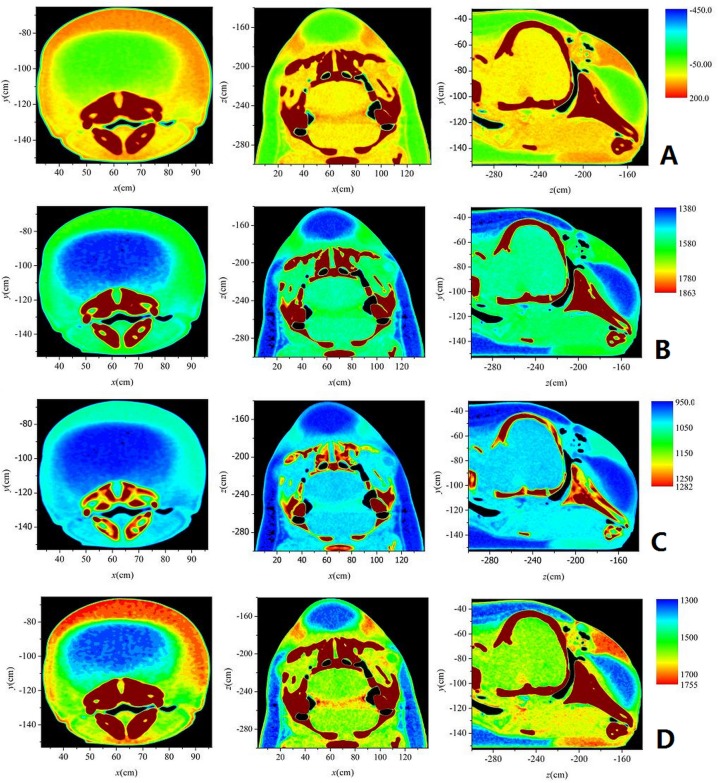
The distributions of HU, sound velocity, density and acoustic impendence of the porpoise’s head at three different sections. (A) HU distribution of the finless porpoise’s head at (left) axial cross section, (middle) coronal cross section and (right) sagittal cross section. (B) Sound velocity distribution of the porpoise’s head at (left) axial, (middle) coronal and (right) sagittal cross sections. (C) Density distribution of the porpoise’s head at (left) axial, (middle) coronal and (right) sagittal cross sections. (D) Acoustic impendence distribution of the porpoise’s head at (left) axial, (middle) coronal and (right) sagittal cross sections.

Using the strong linear relationships of HU vs. sound velocity and HU vs. density, fine distributions of HU, sound velocity, density and acoustic character impendence at three different cross sections (axial, coronal and sagittal in Fig 1) were reconstructed using the same plotting scale, as shown in Fig 5A–5D, where the figures from left to right correspond to the axial, coronal and sagittal cross sections, respectively. Qualitatively consistent patterns were found in HU, sound velocity, density and acoustic impendence.

Standard materials such as room temperature water and air were used for the calibration. From the CT scan, the HU values of water and air were 0 and—1000, respectively, which were consistent with the values in the HU standard. The sound velocity and density of the water calculated from Eqs (3) and (4) were 1501.4 m/s and 1.0008 g/cm^3^, giving errors of 1.12% and 0.3% with respect to the data in the literature (1484 m/s and 0.99754 g/cm^3^), indicating that reliable estimations can be made based on CT imaging. The results of the statistical analysis of HU, sound velocity, density and acoustic impedance in the different structures in the porpoise head are shown in Table 1. From Table 1, the mean values of HU, sound velocity, density and acoustic impendence of the connective tissue are highest, followed by muscle, mandibular fat, melon and blubber. These parameters are significantly different between all tissue types (p<0.001). For HU, the post hoc Dunn’s test shows that connective tissue has significantly higher HU than blubber, muscle, melon, and mandibular fat (p<0.001) and that muscle has significantly higher HU values than blubber, melon, and mandibular fat (p<0.001). Blubber has significantly lower HU values than melon and mandibular fat. However, the HU of the melon tissues are not significantly different from those of mandibular fat (p>0.05).

**Table 1:** Statistical analysis of acoustic parameters of the main tissues of the neonate finless porpoise’s head, including blubber, connective tissue, muscle, melon, and mandibular fat.

	Melon	Blubber	Muscle	Mandibular Fat	Connective Tissue	p value
**Hounsfield Unit**	-63.3 (24.4)	-68 (19.9)	9.6 (7.4)	-59.7 (30.1)	55.8 (20.1)	0.001
**Sound velocity (m/s)**	1426.8 (28.7)	1421.3 (23.4)	1512.6 (8.7)	1431.1 (35.4)	1567.0 (23.6)	0.001
**Density (kg/m** ^**3**^ **)**	969.2 (12.2)	966.8 (9.9)	1005.6 (3.7)	970 (15.0)	1028.7 (10.0)	0.001
**Acoustic impedance (103 Pa•s/m)**	1383.2 (45.4)	1374.3 (36.9)	1521.1(14.4)	1390.1 (52.3)	1612.2 (40.3)	0.001

The numbers in parentheses represent standard deviations.

The sound velocity of the connective tissue is significantly higher than for blubber, muscle, melon, and mandibular fat (p<0.001), and the sound velocity of muscle is significantly higher than those of blubber, melon, and mandibular fat (p<0.001). Blubber has significantly lower sound velocity than melon and mandibular fat. However, the values for the melon are not significantly different from the mandibular fat (p>0.05). These results are consistent with that of the Cuvier’s beaked whale study by Soldevilla et al. [19].

The density of the connective tissue has a significantly higher value than those of blubber, muscle, melon, and mandibular fat (p<0.001), and muscle has significantly higher density than those of blubber, melon, and mandibular fat (p<0.001). Blubber has significantly lower density than the melon and mandibular fat, but the density of the melon is not significantly different from the mandibular fat (p>0.05).

The acoustic impedance is significantly higher for the connective tissue than the blubber, muscle, melon and mandibular fat (p<0.001), and the acoustic impedance of the muscle is higher than for blubber, melon and mandibular fat (p<0.001). There is significantly lower acoustic impedance for blubber than melon and mandibular fat; however, there is no significant difference between melon and mandibular fat (p>0.05).

## Discussion

Previous studies on dolphin biosonar modeling have found that the acoustic properties of the dolphin’s forehead affected the propagation of sounds through the forehead [12, 13, 18, 22]. In this study, computed tomography technology combined with the acoustic properties measurements of tissues were employed to reconstruct acoustic properties of the neonate finless porpoise’s head. The total number of slices in the CT scan of Fig 5 was 140, much larger than that used in ultrasound velocimeter measurement, indicating the higher resolution of CT images. Because the nasal sacs are contained in soft tissue and there is not enough air in the sacs of a dead animal, the entire boundary of each of the sacs is only an approximation to those of a living animal. Nevertheless, the basic structure of the nasal system can be found in the CT images (Fig 5). The vestibular sac, premaxillary sac and nasal plug were empty areas with lower HU, sound velocity, density and acoustic impedance values than the surrounding tissues. The highest values of HU, sound velocity, density and acoustic impedance areas were found in the bony structures of the skull. Tissue sample measurements were not as effective and accurate as results obtained with CT measurements.

The values of the measured sound velocity in Fig 3A were not well correlated to those of the measured density in Fig 3B. The difference might be related to different tissue sample sizes applied in sound velocity and density measurements. In the sound velocity measurement, the probe with the diameter 1.5 cm just covered the area of melon (see Fig 2A). So the measured sound velocity was close to the low sound velocity of melon. However, in the density measurement, the sample was cut according to the red lines, which was bigger than the melon area (see Fig 3B), including the muscle, connective tissue etc., and then the measured density was a medium one. Furthermore, based on the results of ultrasound velocimeter measurement in Fig 3 and CT imaging in Fig 1, general relationships of HU vs. sound velocity and HU vs. density were obtained in Fig 4. These linear relationships are the averaged relationships between the measurement values and HU values of the same regions, which include different tissues; thus they do not correspond to any specific tissue. Additionally, because the averaged sound velocity was measured by the sound probe with 3.5 cm radius, the software Mimics 10.1 (Materialise, Belgium) was used to calculate the averaged HU values of the corresponding region (a circle with 3.5 cm radius) in order to obtain their relationship. The same method was used to obtain the relationship between the measured density values and HU values. Thus there will not be inconsistency between the regions of measurement values and that of the HU values. The tissue samples in Fig 3 were cut too large so that some samples contained different tissues. Large tissue samples and bilateral asymmetry/heterogeneity in the tissue experiments made it difficult to perform high spatial resolution measurements and comparison of acoustic properties. In order to achieve this, high accuracy CT imaging (Fig 5) was applied. The slice number in the CT scan was much larger than that used in ultrasound velocimeter measurement, indicating higher resolution and finer structure in CT images.

The frequency of the ultrasound velocimeter used for the sound velocity measurement, which was 3.5 MHz, had no relationship with that of the animals’ signal frequency. The dolphin can produce approximately 150 kHz acoustic signals for echolocation, instead of the 3.5 MHz used to extract sound velocity of the tissues. In the ultrasound velocimeter, the sound velocity of a tissue was obtained when time *t* could be reliably measured according to the equation *c* = *L*/*t*. It is necessary to use a high frequency probe to ensure the accuracy of time measurement for the sound velocity measurement. For the millimeter accuracy of the tissue length *L*, the probe frequency of the ultrasound velocimeter should be as high as megahertz due to the diffraction effect, which is much higher than the frequency of the dolphin’s acoustic signal.

The derived linear relationships between HU and both sound velocity and density in Fig 4 were consistent with previous studies on Indo-pacific humpback dolphins (*Sousa chinensis*) [23] and Cuvier’s beaked whale [19]. Under room temperature conditions, sound velocity in the neonate finless porpoise’s melon, blubber, muscle, mandibular fat and connective tissue were all slightly higher than those of the Cuvier’s beaked whale. Density in the porpoise’s melon, blubber, muscle, mandibular fat and connective tissue were also close to those of the Cuvier’s beaked whale, which suggested that the physical properties of the tissues in these two species might be similar.

**Fig 6:**
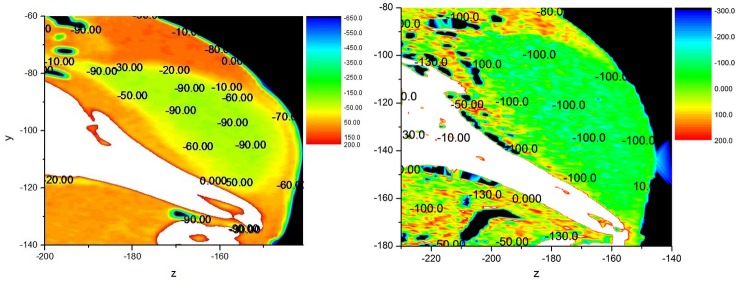
Comparison of the HU values’ distribution on the neonate Yangtze finless porpoise’ head and the one-year-old one’s headat sagittal cross section. The neonate Yangtze finless porpoise’ head is in the left image and the one-year-old one’s head is in the right image.

Finally, several studies have found that sound velocity, density and acoustic impedance of the melon of most dolphin’s increased from inner core to outer layer [10, 11, 24]. However, such a structure was not found in this neonate finless porpoise’s melon, although sound velocity, density and acoustic impedance was significantly lower than those of the surrounding tissues as shown in Fig 5. It is possible that the lipid components in the forehead of the neonate one are not completely laid down until later stages of maturity [25]. To expand on this property further, a specimen of a one-year-old Yangtze finless porpoise scanned in 2011 (unpublished data provided by Zhitao Wang) is shown side by side with the neonate specimen in Fig 6. The distributions of the HU values in both the neonate and the one-year-old porpoises’ heads are shown in the figure. The statistical analysis results of HU values of different tissues in the one-year-old porpoise’s head are shown in Table 2. The results indicate that the HU values of tissues have differences, suggesting that the acoustic properties may change with maturity. The HU values do not show significant increase from the inner core to the outer boundary of melon in the neonate porpoise in Fig 6A, and this increase could barely be found in the one-year-old one. However, it should be noticed that the CT scanner used with the one-year-old porpoise did not have a very high resolution and there were no measured sound velocity or density results. Therefore, it is difficult to make a direct comparison between the neonate and the one-year-old porpoise. Additionally, the one-year-old finless porpoise cannot be considered as a completely adult one; the distribution of acoustic properties from the inner core to the outer boundary in the melons of the older porpoises might have some differences due to the lipid components change with growth. Considering this, this paper focused on the acoustic property reconstruction of a neonate Yangtze finless porpoise’s head based on CT imaging. The comparison between the neonate and adult porpoises might need further study, which could be a potentially important topic.

**Table 2:** Statistical analysis of Hounsfield Unit of the main tissues of the one-year-old finless porpoise’s head, including blubber, connective tissue, muscle, melon, and mandibular fat.

	Melon	Blubber	Muscle	Mandibular Fat	Connective Tissue	p value
**Hounsfield Unit**	-76.7 (43.7)	-76.7 (35.6)	11.7 (13.1)	-70.3 (39.8)	66.3 (18.1)	0.001

The numbers in parentheses represent standard deviations.

## Conclusions

This is the first paper to perform a reconstruction of a neonate finless porpoise’s head based on CT imaging. CT imaging can provide more detailed acoustic properties distribution than direct measurements on the actual tissues. The results showed that the linear relationships between HU and both velocity and density were qualitatively consistent with the results of the Indo-pacific humpback dolphin and Cuvier’s beaked whale. The results can be used for the reconstruction of the acoustic properties of the neonate finless porpoise and also provide the foundation for studying the sound production mechanism and biosonar signals propagation of the neonate finless porpoise. One of the most significant findings of this study is that the melon of the neonate finless porpoise is unlike any other adult odontocetes. The melons of most odontocetes have a low velocity, low-density core with values that increases towards the outer boundary of the melon [24]. No such gradient or profile or either velocity or density were found in the neonate finless porpoise samples of this study.
